# Global burden of mesothelioma attributable to occupational asbestos exposure in 204 countries and territories: 1990–2019

**DOI:** 10.1007/s00432-024-05802-6

**Published:** 2024-05-28

**Authors:** Zhiming Chen, Yikuan Cai, Tongyin Ou, Hu Zhou, Huajie Li, Zhizhi Wang, Kaican Cai

**Affiliations:** Department of Thoracic Surgery, Nanfang Hospital, Southern Medical University, Guangzhou, China

**Keywords:** Mesothelioma, Global burden of disease (GBD), Occupational asbestos exposure

## Abstract

Malignant mesothelioma, a rare and aggressive cancer primarily caused by occupational asbestos exposure, has a poor prognosis. This study leverages the Global Burden of Disease (GBD) 2019 dataset to analyze the burden of mesothelioma linked to occupational asbestos exposure from 1990 to 2019. The analysis includes the number of mesothelioma deaths and disability-adjusted life years (DALYs) attributable to occupational asbestos exposure, focusing on trends in age-standardized mortality rate (ASMR) and age-standardized disability-adjusted life-year rate (ASDR) by year, age, sex, country, region, and Socio-demographic Index (SDI). In 2019, 91.7% of mesothelioma deaths and 85.2% of DALYs were attributable to occupational asbestos exposure, resulting in 26,820 (95% UI 24,312–28,622) deaths and 569,429 (95% UI 509,956–617,484) DALYs. Despite a decline in ASMR and ASDR from 1990 to 2019, the absolute number of deaths and DALYs almost doubled. The United States reported the highest number of mesothelioma deaths, while China had the highest number of DALYs. Age-specific mortality rates and DALYs decreased in the 25–74 age group but increased in the 75+ age group. In conclusion, occupational asbestos exposure remains the primary cause of mesothelioma worldwide, with an increasing number of deaths and DALYs. The highest incidence rates are observed in high-income areas, and rates are rising in low-income areas. It is crucial to raise awareness about the hazards of asbestos to reduce the global burden of mesothelioma linked to occupational exposure.

**Supplementary Information:**

The online version contains supplementary material available at 10.1007/s00432-024-05802-6.

## Introduction

Malignant mesothelioma is a rare and aggressive cancer that can be divided into three distinct histological subtypes: epithelioid, sarcomatoid, and biphasic (Franklin et al. 2016). One study revealed 13 gene mutations associated with mesothelioma pathogenesis, including BAP1 (Rigon et al. 2024) and BRCA2 (Khan et al. 2024). Malignant mesothelioma suffers from limited therapeutic options (Hu et al. 2021) and a poor prognosis (Schulte and Husain 2024) due to long latency (Zhang et al. 2021) and a propensity for treatment resistance (Han et al. 2023). In recent decades, the first-line treatment for unresectable mesothelioma has been platinum-based agents plus pemetrexed (Calabro et al. 2023), with a survival rate of only one year (Courtiol et al. 2019). Recently, hope for prolonging the survival of mesothelioma patients has been on the horizon as new therapies such as immune checkpoint inhibitors have entered clinical trials (Asciak et al. 2021; Calabro et al. 2023; Janes et al. 2021). In 2020, there were 30,870 new mesothelioma cases and 26,278 deaths globally (Sung et al. 2021), with large regional variations.

Due to its high degree of adiabatic and insulating properties, asbestos, a natural mineral, falls into two categories: serpentine (chrysotile) and amphiboles. After inhalation, asbestos fibers remain in the body for a long time, thereby affecting human health. In 2012, asbestos was classified as a Group 1 carcinogen by the WHO (International Agency for Research on Cancer 2024). According to a WHO report in 2018, about 125 million people worldwide are still exposed to asbestos in the workplace. All forms of asbestos are pathogenic (World Health Organization 2014) to humans and may cause diseases such as pulmonary fibrosis (Wilk and Krowczynska 2021), cancer (Straif et al. 2009), (Institute of Medicine Committee on Asbestos and Selected Health Effects 2006; Wronkiewicz et al. 2020; Acheson et al. 1982; Kim et al. 2024) especially cancer of the respiratory system (Henderson and Enterline 1979; Kwak et al. 2022). A GBD study showed that global deaths attributable to occupational asbestos exposure will increase by 2035 (Miao et al. 2024).

Occupational asbestos exposure is the highest risk factor for mesothelioma (Huang et al. 2023), and a meta-analysis found that cessation of occupational asbestos exposure did not reduce the individual risk of mesothelioma (Boffetta et al. 2019). A postmortem case–control study found that asbestos was found in the lungs of 73.7% of mesothelioma decedents, compared to 28% of healthy decedents (Visona et al. 2023). By 1990, asbestos use in most industrialized countries had been reduced by at least 75% from the peak of asbestos consumption (Allen et al. 2018). However, mesothelioma remains a significant cause of death (Hinz and Heasley 2020), because of its extremely poor prognosis and high lethality, (Mutti et al. 2018; Robinson et al. 2005) despite asbestos bans in many countries (Frank and Joshi 2014; Abdel-Rahman 2018). Mesothelioma is highly malignant, and significant changes in the epidemiology of mesothelioma have occurred as a result of increased life expectancy and economic growth. These variables make it a challenge for governments and healthcare organizations to intervene in mesothelioma.

The Global Burden of Disease (GBD) 2019 collaborative group has collected systematic and updated data on 369 diseases and injuries and 87 associated risk factors from more than 204 countries and regions (GBD 2019 Diseases and Injuries Collaborators 2020). This study analyzes the burden of mesothelioma attributable to occupational asbestos exposure at the global, regional, national, age, and sex levels using the most recent GBD 2019 dataset. We also assessed the correlation between mesothelioma burden and SDI. We calculated the EAPC to quantify long-term trends in the age-standardized rate (ASR). These data can clarify the relationship between mesothelioma burden and occupational asbestos exposure and will facilitate early screening, diagnosis, and the development of rational and effective prevention programs.

## Methods

### Data sources

This study used GBD 2019 as a source of data with the aim of assessing the mesothelioma disease burden, analyzing health trends over time, and aiding in the development of global disease intervention strategies. Data collection utilized the Global Health Data Exchange query tool (http://ghdx.healthdata.org/gbd-results-tool), created and maintained by the Institute for Health Metrics and Evaluation (IHME). During the data search, we used "mesothelioma" as the keyword, "occupational asbestos exposure" as the risk factor, "death, DALYs" as the measurement value, "1990–2019" as the year range, and “number, percent, and rate” as the metrics. The number of deaths, DALYs, age-standardized mortality rates (ASMR), and age-standardized DALY rates (ASDR) of mesothelioma cases linked to asbestos exposure was gathered, broken down by year, age, region, and country. The socio-demographic indices (SDI) categorize these data, which span 204 countries and territories, into five classes. We also divided the world into 21 regions, taking into account epidemiologic similarities and geographic proximity. For age distribution, we divided the population into 15 age categories by 5-year age groups to examine age patterns in mortality and DALYs.

### Definitions

For the definition of occupational asbestos exposure-mesothelioma as a risk-outcome pair, GBD 2019 used the World Cancer Research Fund's criteria to determine it (Murray et al. 2020). DALY is a key indicator for quantifying the burden of disease, which represents the sum of years lost due to failure to meet life expectancy and the number of years lived with disability (GBD 2019 Stroke Collaborators 2021). The modeling scheme used to estimate DALYs in GBD 2019 has been described in detail (Murray et al. 2020; GBD 2019 Diseases and Injuries Collaborators 2020). In the GBD 2019 database, the SDI is a composite assessment of regions based on their income levels, fertility rates, and educational attainment. It scores range from 0 to 1, reflecting the overall level of socio-economic status of a region.

### Statistical analyses

We used 95% confidence intervals to estimate deaths, DALYs, ASDR, and ASMR to quantify the burden of mesothelioma attributable to occupational asbestos exposure. To eliminate differences in the age structure of the population, we used population attributable fractions to assess ASDR and ASMR (GBD 2016 Occupational Risk Factors Collaborators 2020). The age-standardized ratio (ASR) is calculated by adding the product of the age-specific ratio for each age group and the standard population proportion for that age group. This allows the different populations to be transformed into standardized populations, thereby eliminating the confounding effects of differences in age structure between the populations being compared. The formula for the ASR is as follows:

$$ASR=\frac{{\sum }_{i=1}^{A}{a}_{i}{w}_{i}}{{\sum }_{i=1}^{A}{w}_{i}}\times 100000$$

wherein i denotes age group, a denotes age-specific rate in the age group, w denotes number of people or weight in the age group, and A denotes total number of age groups.

In epidemiology, the age-adjusted percentage change (EAPC) represents the average percentage change after adjusting for age. By analyzing the relationship between the ASR and changes over time, EAPC allows for the examination of time trends across populations with varying age structures. EAPC was calculated using the following formula: y = α + β x + ε, y = ln(ASR), x = calendar year, and ε = error term. EAPC = 100 (exp (β)-1), and the 95% confidence interval (CI) for EAPC was obtained through a linear regression model. If both the EAPC and lower 95% CI for ASR were > 0, an upward trend was considered; if both the EAPC and upper 95% CI for ASR were < 0, a downward trend was considered; and the remaining results were considered stable over time. We used the Pearson test to assess the association between EAPC, ASR, and HDI. Hierarchical cluster analysis categorized countries and territories around the globe into five groups based on the temporal trends of ASDR and ASMR: (a) Remained stable, (b) Minor increase, (c) Significant increase, (d) Significant decrease, and (e) Minor decrease. All statistics were analyzed using the R program (version 4.2.3).

## Results

### Global trends of mesothelioma attributable to occupational asbestos exposure

**Fig. 1:**
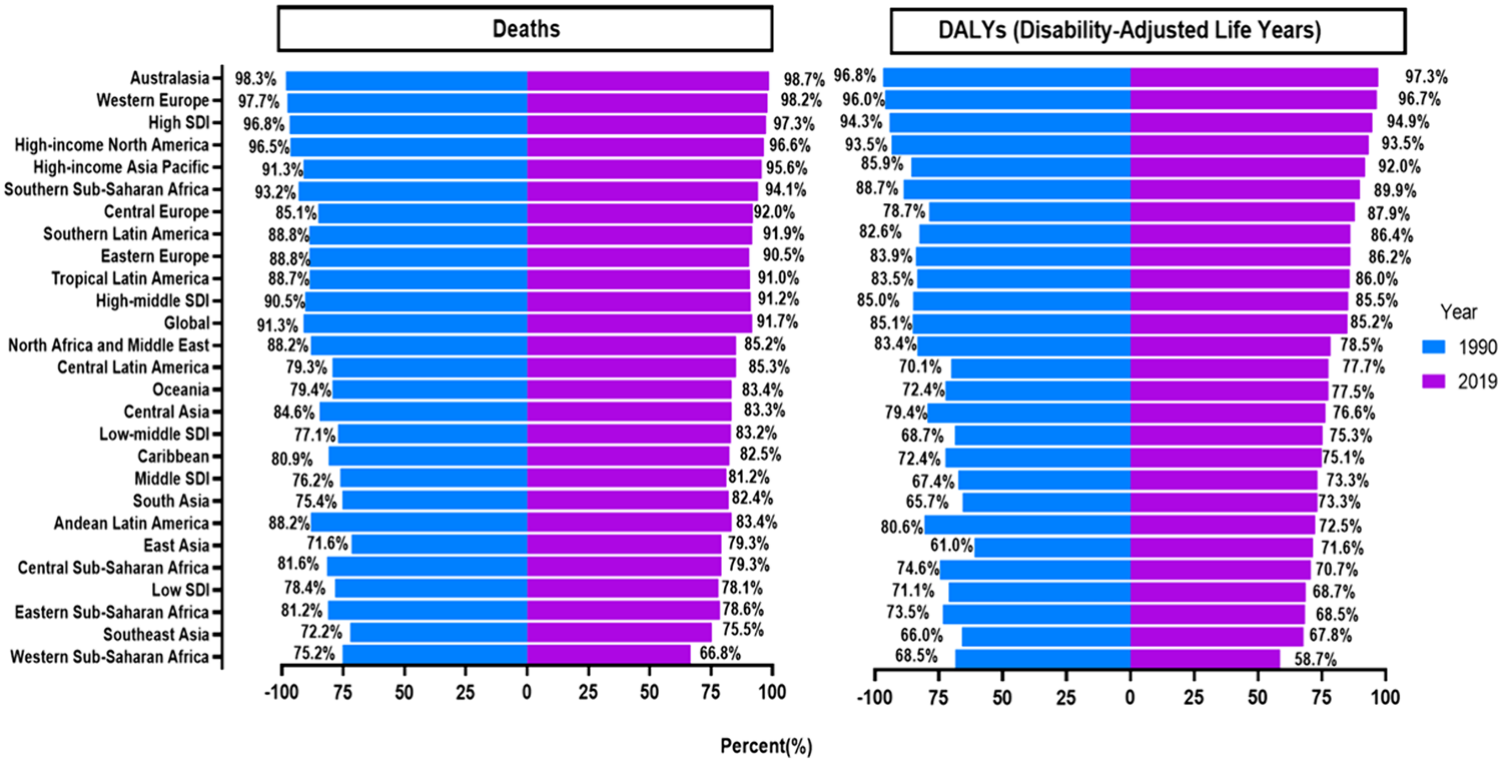
Proportion of mesothelioma deaths and DALYs attributable to occupational asbestos exposure globally and in 26 GBD regions in 1990 and 2019. DALYs, disability-adjusted life-years; GBD, Global Burden of Disease Study

Occupational asbestos exposure has been identified as a significant contributing factor to the incidence of mesothelioma. Our analysis reveals that a substantial burden of mesothelioma cases, approximately 91.7% of deaths and 85.2% of DALYs, can be attributed to occupational asbestos exposure (Fig. 1). In 2019, there were 26,820 (95% UI 24,312–28,622) mesothelioma deaths and 569,429 (95% UI 509,956–617,484) DALYs attributable to occupational asbestos exposure. Mesothelioma deaths and DALYs associated with occupational asbestos exposure have trended upward from 1990 to 2019, with increases of approximately 90% and 70% compared to those of 1990.

In 2019, the ASMR was 0.33 per 100,000 (95% UI 0.3–0.36) and the ASDR was 6.91 per 100,000 (95% UI 6.18–7.48). Both rates showed a decreasing trend between 1990 and 2019, with EAPCs of −0.22% (95% CI −0.29, −0.16) and −0.54% (95% CI −0.6, −0.49), respectively (Table 1). In addition, trends in the number of deaths and DALYs over time were observed in four different age subgroups: there was a decrease in both the number of deaths and DALYs in the 20–64 age group, with a corresponding increase in the over-65 age group (Additional File 1).

**Table 1:** Global burden of Mesothelioma in 1990 and 2019 for both sexes and all locations, with EAPC

Characteristics	1990				2019				EAPC (1990–2019)	
	Deaths cases	ASMR per 100 000	DALYs	ASDR per 100 000	Deaths cases	ASMR per 100 000	DALYs	ASDR per 100 000	ASMR	ASDR
	No. (95% UI)	No. (95% UI)	No.(95% UI)	No. (95% UI)	No. (95%UI)	No. (95% UI)	No. (95% UI)	No. (95% UI)	No. (95% CI)	No. (95% CI)
Global	14,044 (12,483–15,700)	0.37 (0.33–0.42)	332,710 (286,056–383043)	8.24 (7.19–9.34)	26,820 (24,312–28,622)	0.33 (0.3–0.36)	569,429 (509,956–617484)	6.91 (6.18–7.48)	−0.22% (−0.29–0.16)	−0.54% (−0.6–0.49)
** Sex**										
Male	9534 (8865–10,736)	0.6 (0.55–0.66)	220,821 (203,738–253724)	12.19 (11.28–13.88)	19,785 (18,436–21,180)	0.56 (0.52–0.59)	412,876 (382,236–450,034)	10.79 (10–11.72)	0% (−0.09–0.09)	−0.28% (−0.36–0.19)
Female	4510 (3088–5908)	0.21 (0.15–0.28)	111,888 (69,970–157267)	5.11 (3.27–7.04)	7034 (4933–7967)	0.16 (0.11–0.18)	156,553 (105,454–183091)	3.61 (2.42–4.25)	−1% (−1.04–0.95)	−1.31% (−1.35–1.27)
** SDI region**										
High SDI	8232 (7640–9035)	0.77 (0.72–0.85)	174,811 (162,020–192704)	16.84 (15.6–18.54)	14,377 (13,185–15,193)	0.72 (0.66–0.76)	262,335 (243,964–276,666)	14.04 (13.08–14.86)	−0.10% (−0.15–0.04)	−0.57% (−0.63–0.51)
High-middle SDI	3349 (2862–4018)	0.32 (0.27–0.38)	85,680 (71,342–113,175)	7.76 (6.52–9.99)	5859 (5169–6341)	0.29 (0.25–0.31)	132,591 (116,761–146,313)	6.52 (5.73–7.27)	−0.28% (−0.39–0.17)	−0.65% (−0.78–0.53)
Middle SDI	1320 (1046–1703)	0.13 (0.11–0.17)	38,628 (28,927–51,499)	3.37 (2.62–4.38)	3441 (3022–3878)	0.14 (0.12–0.16)	90,425 (77,128–103,896)	3.43 (2.94–3.94)	0.31% (0.19–0.43)	0.11% (−0.02–0.24)
Low-middle SDI	794 (500–1220)	0.14 (0.09–0.21)	23,184 (13,265–37,600)	3.47 (2.12–5.43)	2382 (1892–2982)	0.18 (0.15–0.22)	63,423 (48,031–82058)	4.39 (3.4–5.61)	1.13% (1.04–1.23)	1.03% (0.92–1.15)
Low SDI	342 (131–803)	0.15 (0.06–0.35)	10,222 (3407–22742)	3.8 (1.39–8.79)	746 (386–1554)	0.15 (0.08–0.32)	20,341 (9609–42656)	3.63 (1.84–7.62)	0.06% (−0.05–0.18)	−0.27% (−0.4–0.14)
** GDB regions**										
Andean Latin America	74 (41–102)	0.37 (0.21–0.5)	1963 (993–2882)	9 (4.73–12.86)	99 (71–132)	0.18 (0.13–0.24)	2263 (1536–3158)	3.99 (2.73–5.55)	−3.68% (−4.23–3.12)	−4.13% (−4.76–3.5)
Australasia	424 (371–511)	1.78 (1.56–2.13)	9184 (8054–11000)	39.06 (34.35–46.77)	903 (820–983)	1.72 (1.57–1.88)	16,157 (14,731–17,601)	32.53 (29.69–35.53)	0.05% (−0.08–0.18)	−0.62% (−0.77–0.46)
Caribbean	42 (34–53)	0.16 (0.13–0.2)	1177 (880–1661)	4.33 (3.33–5.96)	77 (59–100)	0.15 (0.11–0.19)	2076 (1485–2903)	3.98 (2.84–5.61)	−0.50% (−0.65–0.35)	−0.49% (−0.64–0.34)
Central Asia	101 (48–228)	0.21 (0.1–0.45)	3380 (1358–8858)	6.36 (2.76–15.54)	146 (98–214)	0.2 (0.14–0.29)	4305 (2717–6763)	5.2 (3.34–7.86)	−0.86% (−1.2–0.52)	−1.61% (−2.01–1.21)
Central Europe	232 (206–297)	0.16 (0.14–0.2)	5983 (5226–7567)	3.99 (3.48–5.06)	551 (469–633)	0.26 (0.22–0.3)	12,796 (10,849–14749)	6.34 (5.32–7.35)	2.36% (2.16–2.57)	2.16% (1.96–2.36)
Central Latin America	133 (112–150)	0.16 (0.14–0.18)	3712 (3076–4323)	4.1 (3.42–4.68)	448 (374–526)	0.19 (0.16–0.22)	11,369 (9326–13,590)	4.68 (3.85–5.56)	0.90% (0.7–1.1)	0.73% (0.53–0.94)
Central Sub-Saharan Africa	47 (13–140)	0.22 (0.07–0.66)	1407 (346–4100)	5.45 (1.46–16.33)	101 (32–295)	0.2 (0.07–0.57)	2883 (839–8703)	4.83 (1.55–14.27)	−0.37% (−0.48–0.26)	−0.57% (−0.69–0.46)
East Asia	859 (603–1297)	0.1 (0.08–0.15)	23,681 (15,323–37,608)	2.51 (1.7–3.87)	2297 (1843–2759)	0.11 (0.09–0.13)	59,186 (46,378–72,397)	2.74 (2.14–3.35)	1.10% (0.71–1.49)	1.24% (0.81–1.67)
Eastern Europe	683 (448–1266)	0.25 (0.16–0.48)	19,551 (11,623–45,287)	7.07 (4.07–16.95)	871 (660–1119)	0.26 (0.2–0.34)	22,932 (17,197–31,635)	7.24 (5.28–10.63)	−0.46% (−0.85–0.07)	−0.80% (−1.27–0.33)
Eastern Sub-Saharan Africa	152 (39–482)	0.21 (0.06–0.68)	4426 (999–13,518)	5.27 (1.32–16.58)	307 (99–979)	0.21 (0.07–0.65)	8268 (2443–26,622)	4.7 (1.51–15.06)	−0.19% (−0.3–0.07)	−0.56% (−0.69–0.43)
High-income Asia Pacific	574 (523–668)	0.29 (0.26–0.34)	13,041 (11,891–14,904)	6.33 (5.77–7.27)	1728 (1533–1866)	0.36 (0.32–0.38)	30,391 (27,754–32,637)	7.13 (6.56–7.66)	1.36% (1.1–1.61)	1.01% (0.72–1.31)
High-income North America	2421 (2254–2620)	0.66 (0.62–0.72)	48,706 (45,715–52,586)	13.94 (13.1–15.04)	3701 (3379–3954)	0.56 (0.51–0.6)	65,891 (60,560–70589)	10.34 (9.49–11.1)	−0.78% (−0.91–0.66)	−1.37% (−1.54–1.2)
North Africa and Middle East	403 (291–611)	0.23 (0.17–0.34)	11,913 (8172–19,141)	6.19 (4.39–9.6)	805 (614–1067)	0.19 (0.15–0.25)	21,332 (15,697–28,411)	4.57 (3.44–6.05)	−0.72% (−0.86–0.58)	−1.22% (−1.38–1.07)
Oceania	5 (3–7)	0.16 (0.11–0.23)	146 (91–238)	4.16 (2.73–6.42)	15 (10–24)	0.22 (0.15–0.32)	475 (275–777)	5.7 (3.54–8.81)	1.47% (1.31–1.64)	1.51% (1.33–1.69)
South Asia	698 (421–1191)	0.13 (0.09–0.22)	19,288 (10,782–34793)	3.17 (1.88–5.46)	2285 (1744–3042)	0.17 (0.13–0.22)	57,413 (42,559–80,135)	3.95 (2.97–5.41)	0.88% (0.77–0.98)	0.79% (0.66–0.93)
Southeast Asia	314 (231–427)	0.11 (0.09–0.15)	11,332 (7326–16,292)	3.46 (2.48–4.79)	721 (541–897)	0.12 (0.09–0.14)	21,914 (15,391–28,519)	3.17 (2.27–4.06)	−0.22% (−0.43–0.01)	−0.88% (−1.16–0.6)
Southern Latin America	116 (104–141)	0.25 (0.23–0.31)	2916 (2604–3634)	6.27 (5.6–7.8)	278 (233–303)	0.33 (0.28–0.36)	6380 (5345–7072)	7.85 (6.59–8.71)	1.63% (1.33–1.93)	1.43% (1.13–1.74)
Southern Sub-Saharan Africa	158 (116–202)	0.6 (0.44–0.76)	4168 (3060–5404)	14.47 (10.71–18.67)	339 (266–384)	0.64 (0.5–0.72)	8280 (6647–9450)	14.37 (11.48–16.26)	0.01% (−0.54–0.55)	−0.32% (−0.94–0.3)
Tropical Latin America	343 (269–435)	0.38 (0.3–0.46)	10,624 (7980–15725)	10.11 (7.88–13.55)	884 (739–1007)	0.37 (0.31–0.42)	23,641 (19,454–27,995)	9.42 (7.72–11.19)	0.08% (−0.05–0.21)	−0.08% (−0.21–0.06)
Western Europe	6173 (5622–6830)	1.05 (0.96–1.15)	133,361 (121,655–146,331)	23.71 (21.62–25.97)	10,123 (9253–10,689)	1.07 (0.98–1.13)	187,208 (173,413–197,949)	21.68 (20.19–23.01)	0.27% (0.18–0.36)	−0.19% (−0.24–0.14)
Western Sub-Saharan Africa	91 (41–165)	0.1 (0.05–0.19)	2750 (1090–4849)	2.74 (1.17–4.94)	141 (79–245)	0.07 (0.05–0.13)	4270 (2160–7363)	1.91 (1.06–3.36)	−1.56% (−1.85–1.27)	−1.72% (−2.02–1.41)

### Regional trends in mesothelioma attributable to occupational asbestos exposure

**Fig. 2:**
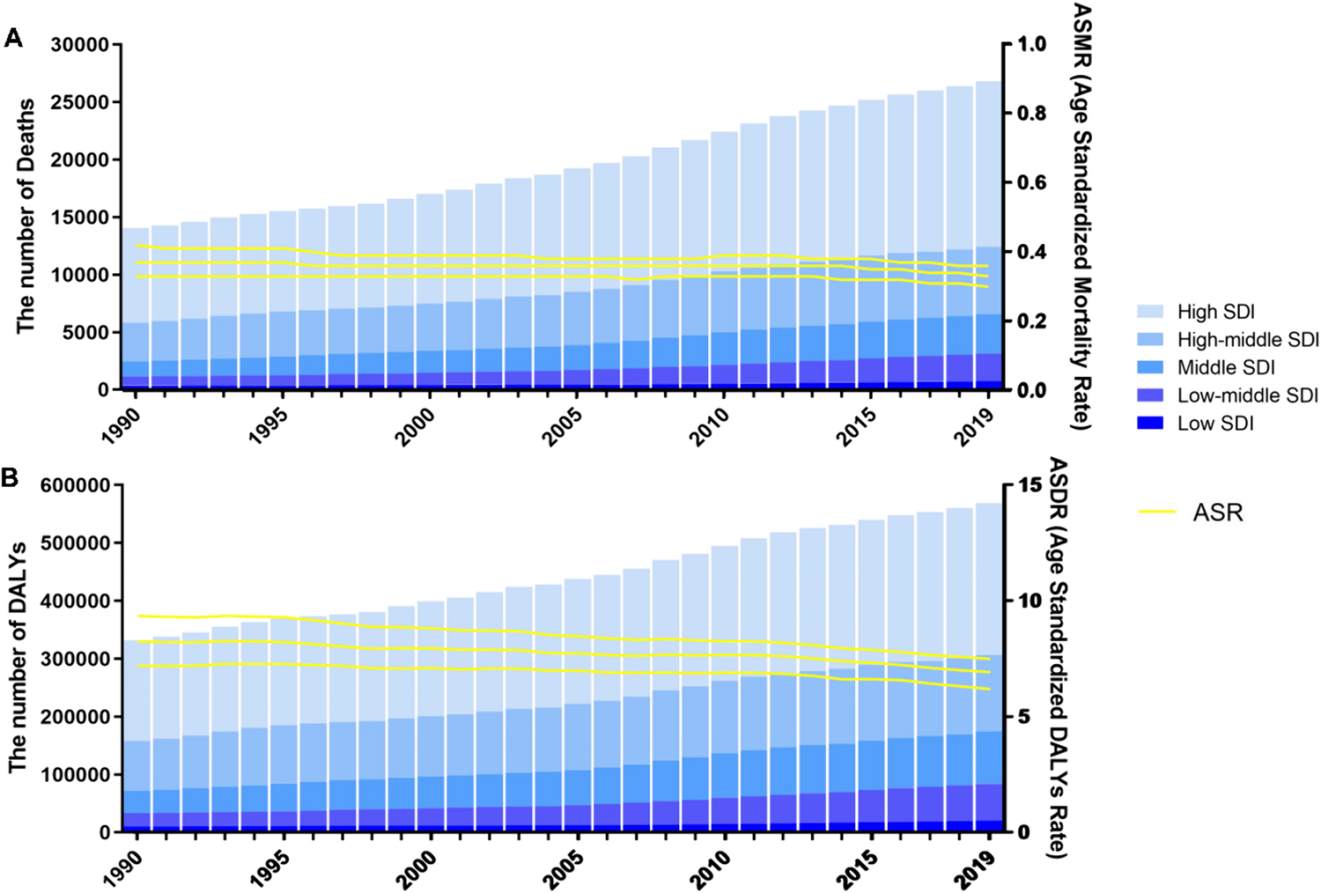
Number and rate of mesothelioma deaths (**a**) and DALYs (**b**) attributable to occupational asbestos exposure between 1990 and 2019 according to the SDI. The bars represent the number of mesothelioma deaths (**a**) and DALYs (**b**) attributable to occupational asbestos exposure colored by SDI level. The line represents the mean ASMR (**a**) and ASDR (**b**) (per 100,000) attributable to occupational asbestos exposure at the global level. The shaded area represents the 95% UI for the mean rate. ASMR, age-standardized mortality rate; DALYs, disability-adjusted life-years; ASDR, age-standardized DALY rate; SDI, socio-demographic index; UI, uncertainty interval

For SDI regions, the High SDI region had the highest number of mesothelioma deaths (14,377, 95% UI 13,185–15,193) and DALYs (262,335, 95% UI 243,964–276,666) related to occupational asbestos exposure in 2019, both accounting for more than 45% of the global total. Also, the high SDI region carried the highest ASMR and ASDR in 2019, at 0.72 per 100,000 (95% UI 0.66–0.76) and 14.04 per 100,000 (95% UI 13.08–14.86), respectively. It was noteworthy that both ASMR and ASDR in High SDI and High-middle SDI areas showed a decreasing trend from 1990 to 2019, with the latter declining even more. On the contrary, ASMR and ASDR in Low-middle SDI regions demonstrated a significant upward trend (Table 1). In addition, the number of mesothelioma deaths and DALYs attributable to occupational asbestos exposure increased in all SDI areas (Table 1). In addition, the number of deaths and DALYs from mesothelioma attributable to occupational asbestos exposure increased significantly in all regions (Fig. 2A, B).

In terms of geographic regions, Western Europe, High-income North America, East Asia, and South Asia have borne the heaviest burden for three decades and have accounted for more than 68 percent of world deaths. However, the highest ASMR and ASDR occurred in Australasia. From 1990 to 2019, ASMR and ASDR declined most significantly in Andean Latin America, with EAPCs of −3.68% (95% CI −4.23, −3.12) and −4.31% (95% CI −4.76, −3.5), respectively. The fastest growth rate was observed in Central Europe, with EAPCs all over 2% (Table 1).

Between 1990 and 2019, the percentage of mesothelioma deaths and DALYs attributable to occupational asbestos exposure was highest in Australasia, followed by Western Europe and High-income North America, all exceeding 90%, while East Asia had the lowest percentage but still exceeded 60% (Fig. 1).

### Countries and cluster analyses in mesothelioma

**Fig. 3:**
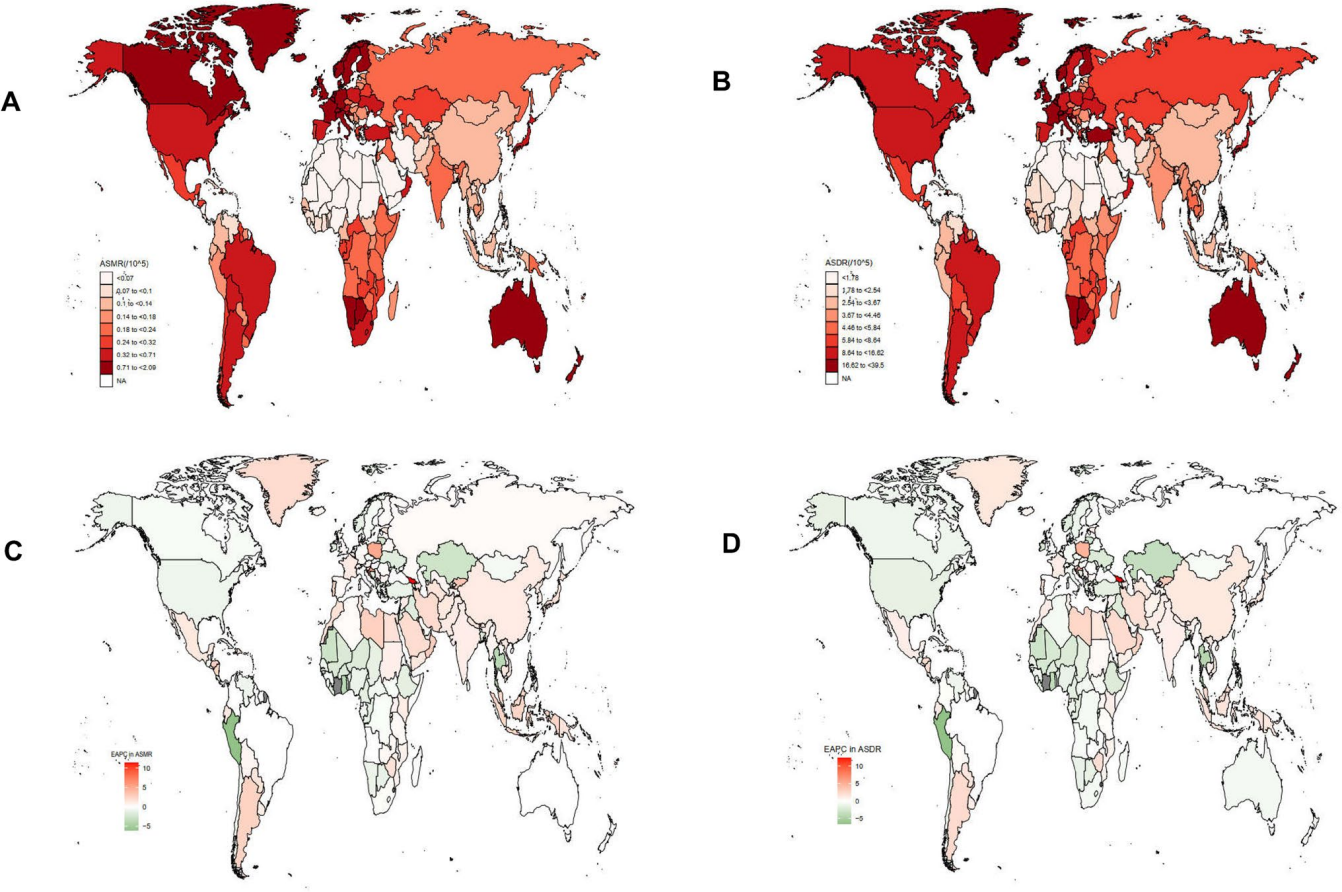
The spatial distribution of the mesothelioma ASMR (**a**) and ASDR (**b**) attributable to occupational asbestos exposure in 2019, and the EAPC in mesothelioma ASMR (**c**) and ASDR (**d**) attributable to occupational asbestos exposure. ASMR, age-standardized mortality rate; ASDR, age-standardized DALY rate; EAPC, estimated annual percentage change

At the country level, the United States of America ranked first in deaths attributable to occupational asbestos exposure in 2019, followed by the United Kingdom; China ranked first in the number of DALYs attributable to occupational asbestos exposure, followed by the United States of America (Additional File 2: Tables S1, S2). The United Kingdom and the Netherlands rank in the top three for both ASMR and ASDR in 2019 (Fig. 3A, B, Additional File 2, Tables S3, S4). In addition, Georgia was the fastest-growing country in ASMR and ASDR from 1990 to 2019, with EAPCs of 11.3% (95% CI 9.12, 13.53) and 12.28% (95% CI 9.94, 14.67), respectively. The fastest decrease in ASMR and ASDR occurred in Peru, with EAPCs −5.98% (95% CI −6.77, −5.18) and −6.52% (95% CI −7.4, −5.63), respectively (Fig. 3C, D, Additional File 2, Tables S5, S6). Percentage changes of mesothelioma deaths and DALYs attributable to occupational asbestos exposure as a proportion of all mesothelioma deaths and DALYs by country were shown in Additional Files 3, 4.

Based on the results derived from cluster analysis, 136 countries (or territories) were categorized in the "remained stable" group, including China, the United Kingdom, and India. 5 countries (or territories) were categorized in the "minor increase" group, including Poland, Kuwait, Croatia, Bahrain, and Qatar. The "minor decrease" group categorized 61 countries (or territories), including the United States and Singapore. It is noteworthy that only Georgia was categorized in the "significant increase" group, and only Peru was categorized in the "significant decrease" group (Additional File 5).

### Global mesothelioma burden attributable to occupational asbestos exposure by age

**Fig. 4:**
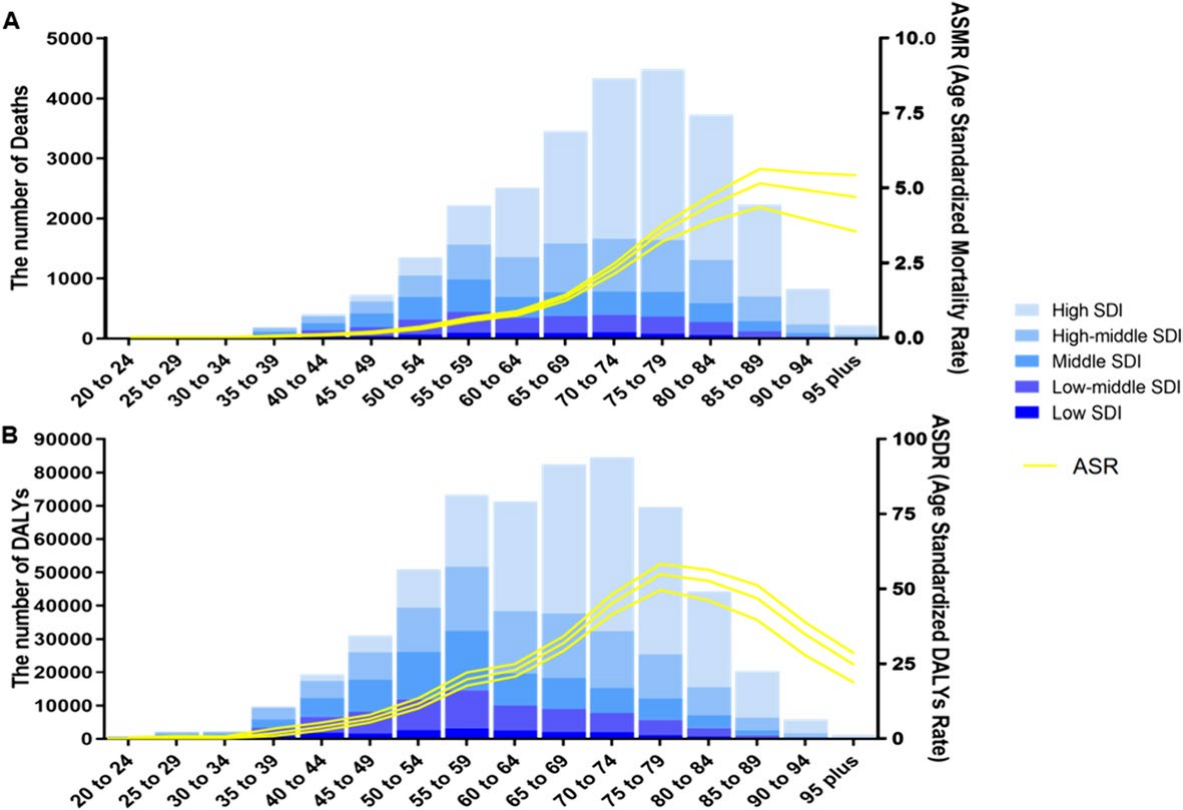
Number and rate of mesothelioma deaths (**a**) and DALYs (**b**) attributable to occupational asbestos exposure by age group and SDI level in 2019. The bars represent the number of mesothelioma deaths (**a**) and DALYs (**b**) attributable to occupational asbestos exposure colored by SDI level. The line represents the mean ASMR (**a**) and ASDR (**b**) (per 100,000) attributable to occupational asbestos exposure at the global level. The shaded area represents the 95% UI for the mean rate. DALYs, disability-adjusted life-years; ASDR, agestandardized DALY rate; SDI, socio-demographic index; UI, uncertainty interval

In 2019, mesothelioma deaths attributable to occupational asbestos exposure increased until 75–79 years of age, then declined in older age categories. Most deaths occurred in the 65–84 age group, with a peak in the 75–79 age group, and more age-specific deaths occurred in the high and high-middle SDI regions compared to the low and low-middle SDI regions (Fig. 4A). Similar to the trend in the number of deaths, the majority of DALYs occur at ages 55–79, with a peak at ages 70–74 (Fig. 4B). Besides, age-specific mortality reached its peak at ages 85–89 and then gradually decreased, while the inflection point for DALYs was at ages 75–79.

**Fig. 5:**
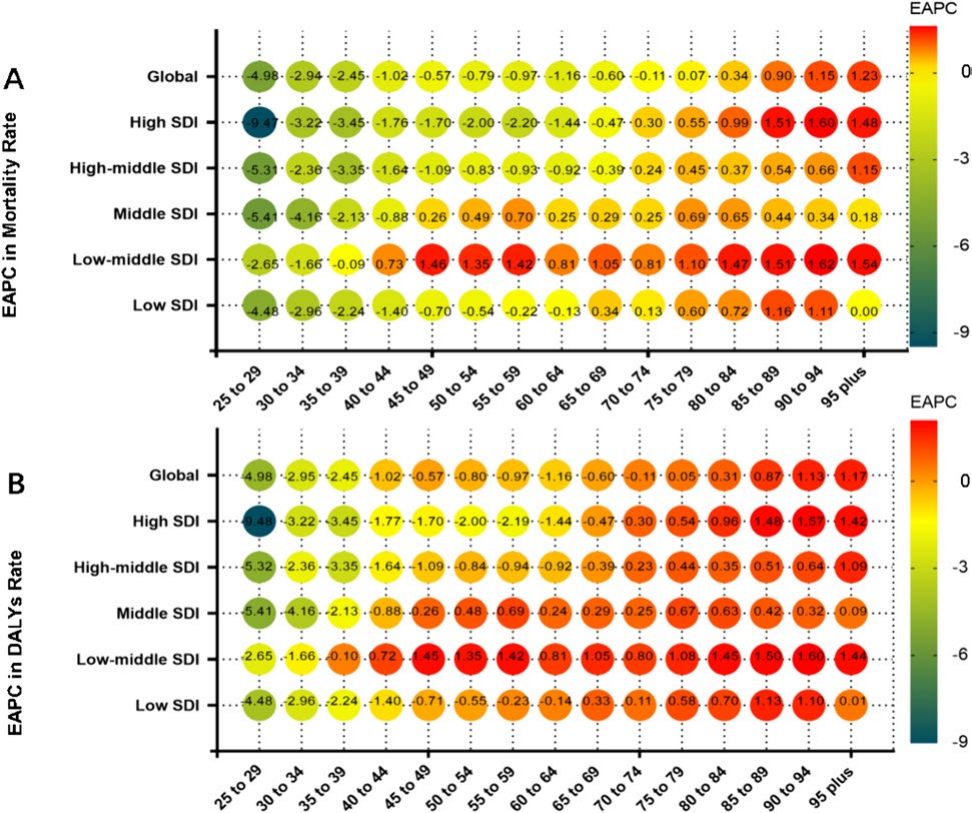
Annual percentage change in mortality (**a**) and DALYs (**b**) between 1990 and 2019 by age group and region. EAPC, estimated annual percentage change; SDI, socio-demographic index; DALYs, disability-adjusted lifeyears

Globally, the mortality rate and DALYs rate have decreased from 1990 to 2019 in the 25–74 age group. However, both rates have increased in the over-75 age group, with a greater increase observed with age. Across all districts and all age groups, High SDI region has the lowest EAPCs for mortality rate and DALYs, which are both less than −9% in the 25–29 age group, and Low-middle SDI region has the highest EAPCs for mortality rate and DALYs, which are about 1.6 in the 90–94 age group. In High SDI and High-middle SDI regions, age-specific mortality and DALYs rates decreased in the 25–69 age group and increased in the 70 + age group, other SDI regions showed the same pattern at different age cutoffs (Fig. 5A, B).

### Factors associated with mesothelioma burden attributable to occupational asbestos exposure

Overall, ASMR and ASDR were correlated with SDI in 2019 for different regions (*R* = 0.529, *p* < 0.001; *R* = 0.519, *p* < 0.001; respectively), with the first inflection point at 0.55 and higher ASMR and ASDR at the second inflection point, which occurred around 0.8 (Additional Files 6A, B). We have also visualized the association of ASMR and ASDR with SDI for each country. Across countries, as SDI increased, ASMR and ASDR increased modestly until SDI was about 0.7, after which they increased sharply as SDI increased. Based solely on SDI, the ASMR and ASDR for the United Kingdom, Australia, the Netherlands, and Lesotho are all much higher than expected (Additional Files 7, 8).

A highly negative correlation was observed between the EAPC in ASDR and ASDR in 1990 across different countries (*R* = −0.23*, p* < 0.001) (Additional File 9). Also, a statistically negative correlation was observed between EAPC in ASMR and ASMR 1990 across different countries (*R* = −0.16*, p* < 0.05) (Additional File 10).

## Discussion

In this study, we systematically analyzed and summarized the epidemiological characteristics of global mesothelioma attributable to occupational asbestos exposure using the latest GBD 2019 data. The results show that occupational asbestos exposure is the leading cause of mesothelioma deaths and DALYs worldwide, accounting for more than 91% and 85% of the total, respectively. Over the past three decades, despite a slight downward trend in ASMR and ASDR for mesothelioma attributable to occupational asbestos exposure globally, the corresponding absolute number of deaths and DALYs has almost doubled. We can attribute this phenomenon to the aging of the population and the increase in the total population base.

In terms of the spatial distribution of mesothelioma attributable to occupational asbestos exposure, High SDI region has a higher proportion of deaths and DALYs. In the interval of SDI 0.7–0.8, ASMR and ASDR showed a significant increase with increasing SDI. In developed countries, the use of asbestos peaked in 1980 at about 4.7 million tons due to its extensive use in industry and construction before its pathogenicity was realized (Virta 2006). The number of mesothelioma cases attributable to occupational asbestos exposure has significantly increased in recent decades due to the latency period of several decades. In addition, with modern advances in immunology and molecular diagnostic techniques (Mukhopadhyay et al. 2024), the diagnostic specificity and ability to differentiate mesothelioma have improved (Burdorf et al. 2007), leading to an increase in the number of reported cases. The increased prevalence of mesothelioma attributable to occupational asbestos exposure began in the late twentieth century in developed nations that had undergone significant economic expansion. Mortality and DALYs emerge relatively late in low- and middle-income countries. Although underdeveloped regions have lower mortality rates and DALYs, the inadequacy of their healthcare infrastructure and the lower use of asbestos may contribute to this phenomenon. A shortage of medical resources may affect timely diagnosis (Chimed-Ochir et al. 2020), availability of treatment, and management of cancer patients. Despite the insignificant growth trend, less developed regions should learn from developed regions' experience and promptly alert themselves to the hazards of asbestos.

In terms of age distribution, mortality and DALYs showed an increasing trend in middle-aged and older age groups. This can be explained by the fact that this group was exposed to asbestos at a young age and developed the disease after a latent period. Advances in biopsy techniques and imaging have also led to increased detection rates (Khan et al. 2024). Notably, the most significant decrease in mortality and DALYs of mesothelioma due to occupational asbestos exposure across all age groups was in the 25–29 age group, with EAPCs even less than −9% in the High SDI region. This may be a function of the earlier asbestos bans enacted in developed countries (Jarvholm and Burdorf 2015). People in the 25–29 age group may never have been exposed to asbestos since birth, so the decline is most pronounced in this age group.

In terms of global asbestos policy, the United Kingdom enacted the world's first asbestos-related regulation in 1931, limiting occupational asbestos exposure for asbestos manufacturers (Jarvholm and Burdorf 2024). On October 23, 2023, the Council of the European Union formally adopted new asbestos rules that should be equal to 0.002 fibers per cm^3^ when counting fibers with a breadth of between 0.2 and 3 µm, or 0,01 fibers per cm^3^ when also counting fibers with a breadth of less than 0.2 µm (Council of the European Union 2023). As of October 28, 2022, 69 countries around the world have enacted bans on asbestos, including the entire EU region (World Health Organization 2014), according to the International Ban Asbestos Secretariat (International Ban Asbestos Secretariat 2022). Since the 1970s, the United States has imposed strict regulatory measures on the use of asbestos, a policy that has led to a significant reduction in the incidence of mesothelioma, thus proving the value of prevention initiatives (Alpert et al. 2020). Nevertheless, the total number of deaths due to mesothelioma has not shown a corresponding trend of decrease due to the increase in the total population, especially the elderly population (Carbone et al. 2019). Australasia has the highest ASMR and ASDR in the world, and the Australian government enacted regulations to ban all types of asbestos use in 2003 (Asbestos and Silica Safety and Eradications Agency 2016; Soeberg et al. 2016). Canada, once the world's largest producer of asbestos, also banned asbestos use completely by the end of 2018 (Jarvholm and Burdorf 2024). However, the risk of asbestos exposure still exists in countries that have banned the use of asbestos completely, and a study by academics in Colombia found that an average of 20% of concrete roofs contain asbestos (Martinez et al. 2024). Large quantities of asbestos-containing materials in building maintenance or demolition operations potentially expose workers to asbestos. On the other hand, the use of asbestos persists in a number of relatively resource-poor countries, despite the fact that bans on the use of asbestos have been implemented in some countries (Freemantle et al. 2022). According to estimated data on global asbestos consumption in 2016, India, a resource-poor country, led the world in asbestos consumption with 308,000 tons (Chen et al. 2019). Therefore, the problem of occupational asbestos exposure should not be ignored in either developed (Han et al. 2022) or developing countries (Chen et al. 2019). Many scholars have discovered methods of classifying (Lee et al. 2024) and harmlessly disposing (Capitani et al. 2024) of asbestos, which has helped to reduce exposure to asbestos.

This study validated the relationship between occupational asbestos exposure and mesothelioma by analyzing the GBD database, which helps to quantify the risk of asbestos exposure and provides targets for occupational health and safety measures. In addition, by analyzing different age groups and different countries, the study will help to raise the public's awareness of self-protection, exposure risk reduction and proactive screening, especially in high-risk industries. These are essential for early screening and diagnosis.

This study used the latest GBD 2019 data to analyze the heavy disease burden of mesothelioma attributable to occupational asbestos exposure and to analyze the effectiveness of interventions, but some limitations remain. First, there is a lack of quantitative indicators of occupational asbestos exposure, and some patients are unaware of their exposure to this risk factor. Second, patient screening is not well established in some less developed countries, which may undermine the accuracy and reliability of our findings. Third, the implementation of policies related to asbestos varies greatly from country to country, so it is difficult to link our findings to the asbestos ban policies well. Fourth, the effects of different types of asbestos were not distinguished in this study or the GBD 2019 database. Finally, the different subtypes of mesothelioma have important prognostic implications, but differentiation data for the different subtypes were not distinguished in GBD 2019 (Dacic 2022).

## Conclusion

The effective implementation of asbestos bans in a few countries tells us that occupational asbestos exposure is a modifiable risk factor for mesothelioma. The intervention aims to reduce the risk of mesothelioma in the population by banning the use of all types of asbestos. Mesothelioma deaths attributable to occupational asbestos exposure are continuing to rise (Andujar et al. 2016), and the crisis in the global healthcare system has not been abated. Therefore, governments, companies, society, health care systems, and individuals should work together to raise awareness of the dangers of occupational asbestos exposure and promote the implementation of policies. The specific implementation of bans varies considerably from country to country. Interventions should be adapted to geographic differences, demographics, and economic development. The ultimate goal is to reduce the burden of mesothelioma attributable to occupational asbestos exposure.

## Supplementary Information

Below is the link to the electronic supplementary material.

Supplementary file1 (PNG 228 KB)

Supplementary file2 (PNG 13 KB)

Supplementary file3 (XLSX 12 KB)

Supplementary file4 (XLSX 18 KB)

Supplementary file5 (XLSX 19 KB)

Supplementary file6 (PNG 43 KB)

Supplementary file7 (PNG 41 KB)

Supplementary file8 (PNG 169 KB)

## Data Availability

The Global Burden of Disease (GBD) Study estimates supporting the conclusions of this article are available from the Institute for Health Metrics and Evaluation (IHME) GBD Results Tool | Global Health Data Exchange, https://vizhub.healthdata.org/gbd-results/. Human Data on the Human Development Index (HDI) was obtained from the United Nations Development Program (https://hdr.undp.org/data-center).
