# Supplementary figures and images for: Global burden of mesothelioma attributable to occupational asbestos exposure in 204 countries and territories: 1990–2019
Source: J Cancer Res Clin Oncol. 2024 May 28;150(5):282. doi: 10.1007/s00432-024-05802-6 (PMC11133219; doi:10.1007/s00432-024-05802-6)

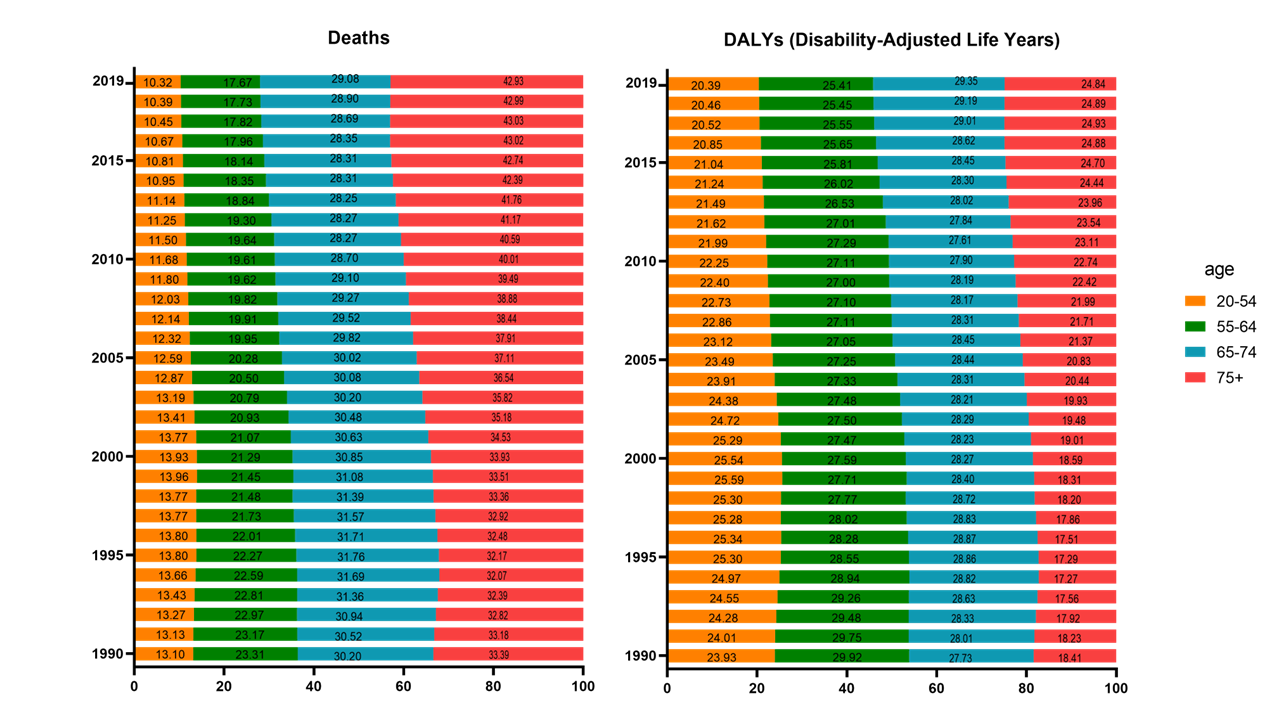

Supplement: Supplementary file 1 — Supplementary file1 (PNG 228 KB) [file 432_2024_5802_MOESM1_ESM.png]

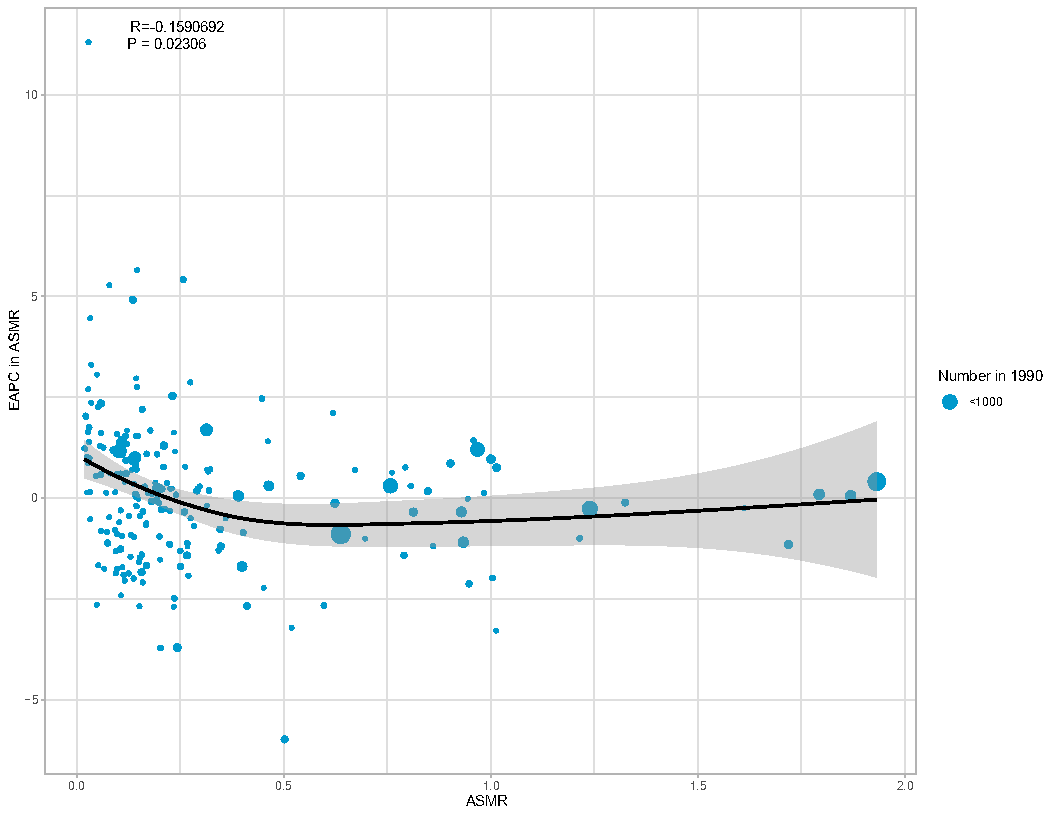

Supplement: Supplementary file 2 — Supplementary file2 (PNG 13 KB) [file 432_2024_5802_MOESM2_ESM.png]

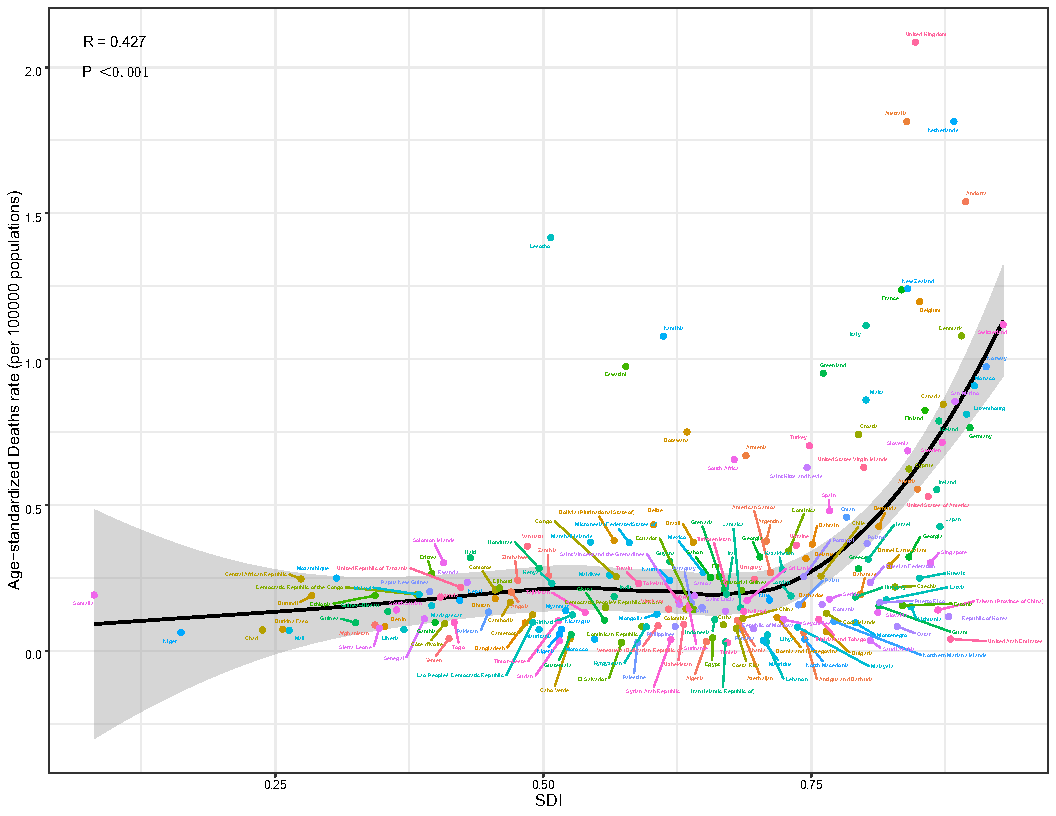

Supplement: Supplementary file 6 — Supplementary file6 (PNG 43 KB) [file 432_2024_5802_MOESM6_ESM.png]

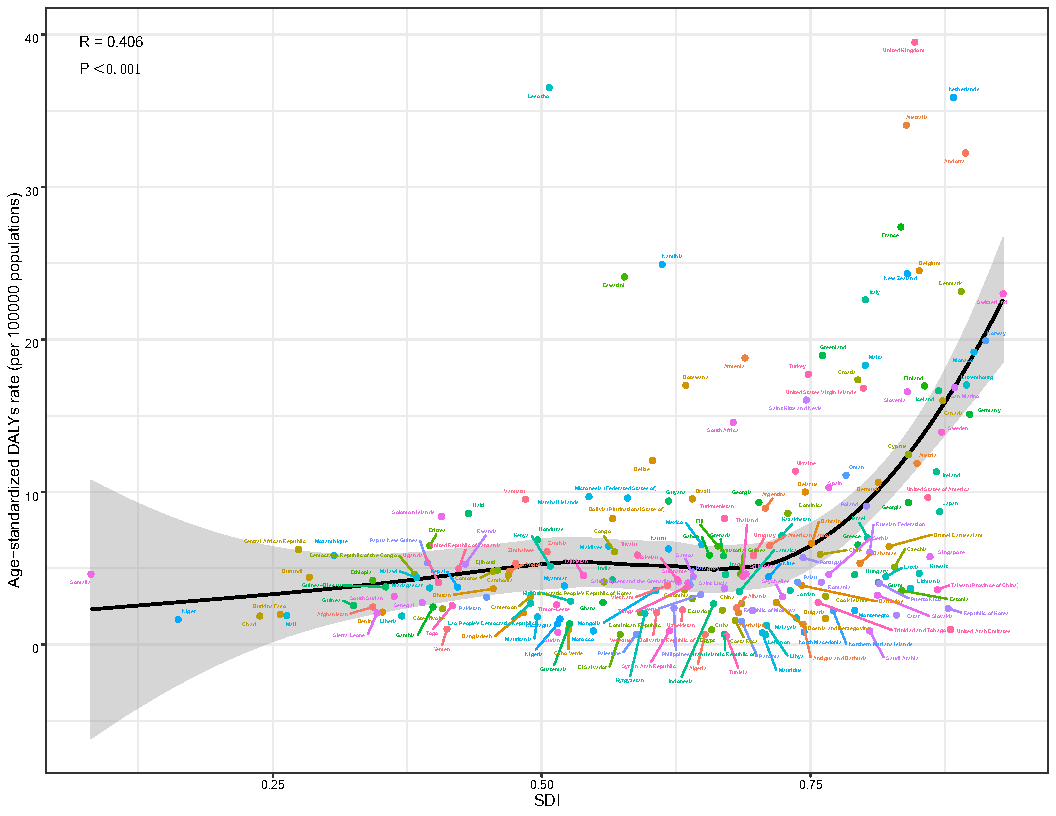

Supplement: Supplementary file 7 — Supplementary file7 (PNG 41 KB) [file 432_2024_5802_MOESM7_ESM.png]

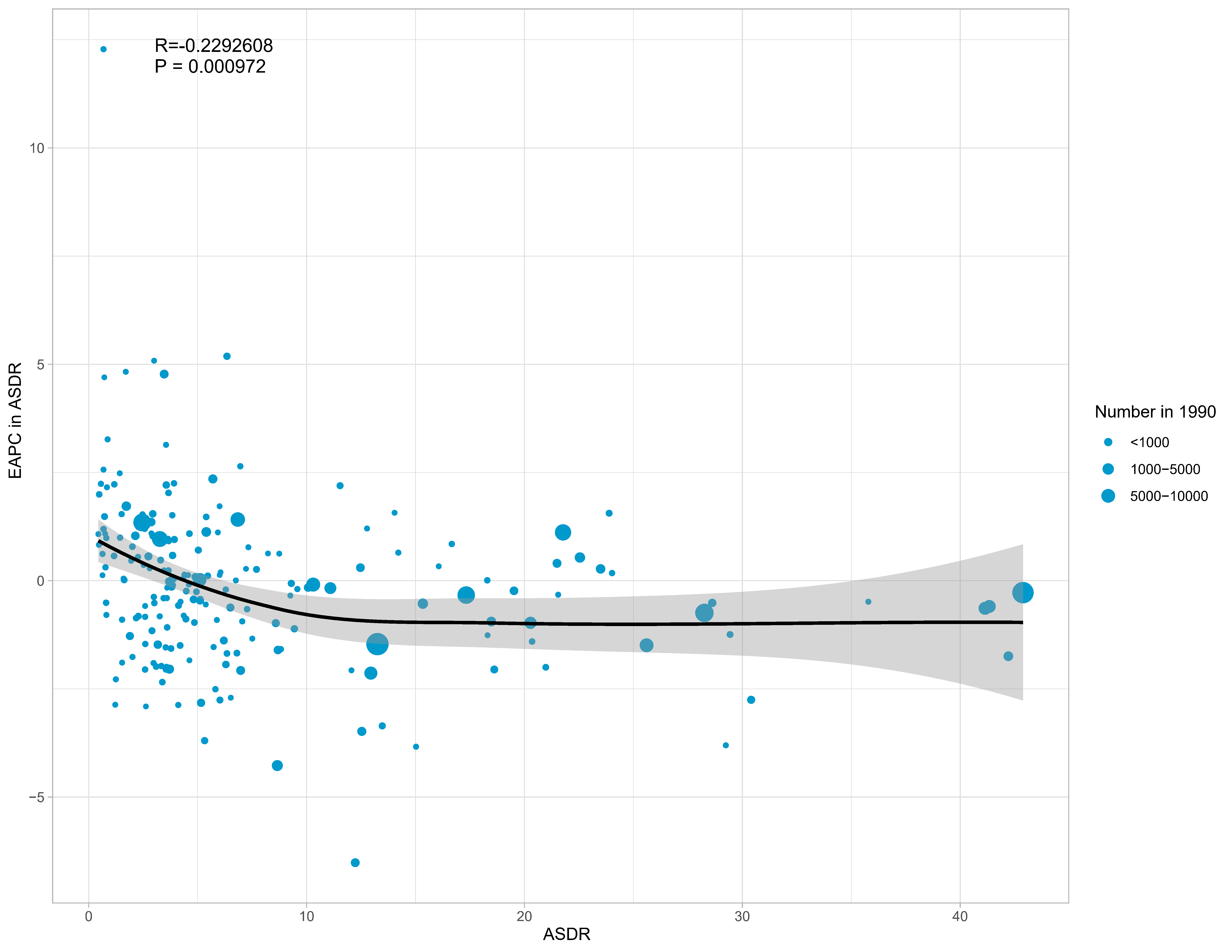

Supplement: Supplementary file 8 — Supplementary file8 (PNG 169 KB) [file 432_2024_5802_MOESM8_ESM.png]
